# Gut microbiome and plasma metabolome alterations in myopic mice

**DOI:** 10.3389/fmicb.2023.1251243

**Published:** 2023-12-21

**Authors:** Hao Li, Shuyu Liu, Keke Zhang, Xiangjia Zhu, Jinhui Dai, Yi Lu

**Affiliations:** ^1^Department of Ophthalmology, Zhongshan Hospital, Fudan University, Shanghai, China; ^2^Eye Institute, Eye & ENT Hospital, Fudan University, Shanghai, China; ^3^NHC Key Laboratory of Myopia (Fudan University), Shanghai, China; ^4^Key Laboratory of Myopia, Chinese Academy of Medical Sciences, Shanghai, China; ^5^Shanghai Key Laboratory of Visual Impairment and Restoration, Shanghai, China

**Keywords:** myopia, gut microbiota, metabonomics, gut barrier function, 16S rRNA

## Abstract

**Background:**

Myopia is one of the most common eye diseases leading to blurred distance vision. Inflammatory diseases could trigger or exacerbate myopic changes. Although gut microbiota bacteria are associated with various inflammatory diseases, little is known about its role in myopia.

**Materials and methods:**

The mice were randomly divided into control and model groups, with the model group being attached-30D lens onto the eyes for 3 weeks. Then, mouse cecal contents and plasma were collected to analyze their intestinal microbiota and plasma metabolome.

**Results:**

We identified that the microbial composition differed considerably between the myopic and non-myopic mice, with the relative abundance of Firmicutes phylum decreased obviously while that of Actinobacteria phylum was increased in myopia. Furthermore, Actinobacteria and Bifidobacterium were positively correlated with axial lengths (ALs) of eyeballs while negatively correlated with refractive diopters. Untargeted metabolomic analysis identified 141 differentially expressed metabolites, and Kyoto Encyclopedia of Genes and Genomes pathway enrichment analysis revealed considerable enrichment mainly in amino acid metabolism pathways. Notably, pathways involved glutamate metabolism including “Glutamine and D-glutamate metabolism” and “Alanine, aspartate and glutamate metabolism” was changed dramatically, which presented as the concentrations of L-Glutamate and L-Glutamine decreased obviously in myopia. Interestingly, microbiome dysbiosis and metabolites alternations in myopia have a disrupting gut barrier feature. We further demonstrated that the gut barrier function was impaired in myopic mice manifesting in decreased expression of Occludin, ZO-1 and increased permeation of FITC-dextran.

**Discussion:**

Myopic mice had obviously altered gut microbiome and metabolites profiles compared to non-myopic mice. The dysbiosis and plasma metabolomics shift in myopia had an interrupting gut barrier feature. Our study provides new insights into the possible role of the gut microbiota in myopia and reinforces the potential feasibility of microbiome-based therapies in myopia.

## 1 Introduction

Myopia, also known as nearsightedness, is a significant global public health concern (Baird et al., 2020). The World Health Organization (WHO) estimates that the global prevalence of myopia will increase by 50% and high myopia (HM) by 10% by 2050 (Holden et al., 2016). Notably, the prevalence of myopia is rapidly increasing in East Asia (Yamada et al., 2010). HM is defined as refraction lesser than −6.00 diopters or an eye axial length (AL) greater than 26 mm, and is a severe form of myopia that can result in blindness threatening complications, such as macular degeneration, cataract, retinal detachment (Morgan et al., 2012).

The gut microbiome is a complex ecosystem of microbes and their genetic entities colonized in the gastrointestinal tract. Intestinal flora plays a vital role in numerous pathophysiological events, such as nutrient metabolism, immune response, and maintenance of the intestinal barrier (Natalini et al., 2022). Normally, the gut microbial composition maintains in a state of homeostasis. However, the balanced composition of gut microbes would be disrupted under the state of illness. The dysbiosis accompanied by incremental pathogens and their associated metabolites, which in turn exacerbate primary diseases (Collins et al., 2022). Numerous studies have reported that alterations in the intestinal microbiota contribute to the pathogenesis of various common diseases, such as inflammatory bowel disease (Loayza et al., 2023), obesity (Patterson et al., 2016), diabetes (Wang D. et al., 2022), neuropsychiatric diseases (Camilleri and Vella, 2022), and cancer (Fernandes et al., 2022).

Recent studies found that microbe composition and their metabolites variation were correlated with eye diseases, suggesting the existence of a gut-eye axis. Gut microbiome is involved in the onset and progression of multiple ocular diseases, including uveitis, ceratitis, age-related macular degeneration and retinal artery occlusion (Napolitano et al., 2021). Metabolomics studies of myopia discover that myopia development respond to biological metabolic changes (Hou et al., 2022). Importantly, gut microbiome-derived metabolites are the vital mediators in interactions between gut microbiota and the host. Moreover, inflammatory diseases could trigger or exacerbate myopic changes (Lin et al., 2016). However, the role of gut microbiota in myopia progression remains unclear. It is necessary to clarify the gut microbiota and metabolic phenotype to understand their role in myopia progression.

In our study, we compared the gut microbiota composition and plasma metabolic phenotype between myopic mice model and controls. We illustrated that myopia shaped the composition of the gut microbiome, and the dysbiosis and disorder of its metabolites may contribute to the deterioration of myopia. Our study sheds light on the potential role of the gut microbiota in myopia and prompts the potential feasibility of microbiome-based therapies in myopia.

## 2 Materials and methods

### 2.1 Experimental animals and husbandry environment

Forty 3-week-old male C57BL/6 mice were purchased from SLAC Laboratory Animal Co. Ltd. The mice were housed in specific pathogen-free conditions with food and water *ad libitum* at 22°C with 40%–60% humidity under 12:12 h light/dark cycle. The experimental procedures involving animals were conducted in accordance with the ARVO Statement for the use of animals in research, and were approved by the Ethics Committee for Animal Studies at the Eye & ENT Hospital of Fudan University.

### 2.2 Mouse model of high myopia

The mouse model of lens-induced high myopia was conducted according to previous study with moderate modification (Zhu et al., 2021). Briefly, mice were attached-30D lens onto the periorbital skin of the eyes to establish the lens-induced high myopia model and the mice wearing plain lens were served as control. Refractive state was measured by infrared photorefractor (Steinbeis Transfer Center, Germany) at the beginning of the study. Mice with anisometropia (≥1D), corneal trauma and lens opacity were excluded. Mice were checked every day to make sure the attachment of lens. After 3 weeks, the axial length of eyeball was imaged with High-resolution 7.0 Tesla MRI (Biospec 70/20 USR, Bruker) and measured with Image J. The refractive state of mice was measured and mice with at least 6.00D myopia shifts compared to control group were used for further experiments.

### 2.3 Fecal and tissue collection

After modeling success, mice were anesthetized by pentobarbitone sodium. Blood samples were collected immediately prior to sacrificing the mice for further metabolomics analyses. Then, mice were euthanized through cervical dislocation on a sterile operating table, the fecal was obtained from the cecal and stored in −80°C. Segments of intestine were collected and fixed in 4% paraformaldehyde or stored in −80°C until use.

### 2.4 Fecal gut microbiota analyses

Total bacterial DNA of mouse cecal content was extracted using QIAamp DNA stool mini kit (Qiagen) according to the manufacturer’s instructions. The concentration of DNA was determined by NanoDrop 2000 (Thermo Scientific). The hypervariable V3–V4 region of the bacterial 16S rRNA was amplified by polymerase chain reaction with primers 338F (5’-ACTCCTACGGGAGGCAGCAG-3′) and 806R (5’-GGACTACHVGGGTWTCTAAT-3′). Purified amplicons were pooled in equimolar amounts and paired-end sequenced (2 × 250 bp) on HiSeq PE250 platform (Illumina) by Personal Biotechnology Co., Ltd. (Shanghai, PR China).

Microbiome bioinformatics was performed using QIIME 22019.4 (Bolyen et al., 2018) and R software (version 4.0.3). Raw data were demultiplexed, quality filtered, denoised, merged and chimera removed using the DADA2 plugin. Non-singleton amplicon sequence variants (ASVs) were aligned with mafft (Katoh et al., 2002) and used to construct a phylogeny with fasttree2 (Price et al., 2010). Chao1, Shannon, Simpson indices were calculated for alpha diversity analysis. For beta-diversity, weighted UniFrac distance was calculated and principal coordinates analysis (PCoA) plots was generated. The significant difference was assessed using PERMANOVA. Linear discriminant analysis effect size (LEfSe) was used to find differentially abundant taxa at different levels between groups (alpha <0.05 and LDA > 2.0).

### 2.5 Untargeted metabolomics

The plasma samples were thawed at 4°C. A total of 200 μL plasma were mixed with 400 μL methanol and vortexed for 60 s. The samples were centrifuged with 12,000×*g* for 10 min at 4°C and then transferred to new tubes. Samples were concentrated to dry in vacuum. Samples were dissolved with 150 μL 2-chlorobenzalanine (4 ppm) 80% methanol solution, and the supernatant was filtered through 0.22 μm membrane to obtain the prepared samples for LC–MS; 20 μL from each sample was mixed to be quality control (QC) samples.

Samples were analyzed by liquid chromatography-mass spectrometry (LC–MS). Briefly, chromatographic separation was accomplished in an Thermo Ultimate 3,000 system equipped with an ACQUITY UPLC^®^ HSS T3 (150 × 2.1 mm, 1.8 μm, Waters) column maintained at 40°C. Gradient elution of analytes was carried out with 0.1% formic acid in water (A) and 0.1% formic acid in acetonitrile (B) or 5 mM ammonium formate in water (C) and acetonitrile (D) at a flow rate of 0.25 mL/min. The injection volume was 2 μL. The gradient was as follows: 0 ~ 1 min, 2% B/D; 1 ~ 9 min, 2% ~ 50% B/D; 9 ~ 12 min, 50% ~ 98% B/D; 12 ~ 13.5 min, 98% B/D; 13.5 ~ 14 min, 98% ~ 2% B/D; 14 ~ 20 min, 2% B-positive model (14 ~ 17 min, 2% D-negative model). The ESI-MSn experiments were carried out based on the Thermo Q Exactive mass spectrometer with the spray voltage of 3.8 kV and − 2.5 kV in positive and negative modes, respectively. The capillary temperature was 325°C. The analyzer scanned over a mass range of m/z 81–1,000 for full scan at a mass resolution of 70,000. Data dependent acquisition MS/MS experiments were performed with HCD scan. The normalized collision energy was 30 eV.

Raw data were converted into mzXML format using Proteowizard software (v3.0.8789). Peaks identification, peaks filtration and peaks alignment were conducted by R (v3.3.2). Partial least square discriminant analysis (PLS-DA) was used to visualize the distribution and the grouping of the samples. To avoid model overfitting, we conducted permutation permutations test. Significant changed metabolites were identified (*p*-value ≤ 0.05 and VIP ≥ 1). Metabolites were annotated using HumanMetabolome Database (HMDB), Metlin, and Bio-ML databases. Metabolites pathway analysis was carried out in metaboanalyst.^1^

### 2.6 Immunofluorescence staining

Intestine tissues fixed in paraformaldehyde were embedded in paraffin, and cut into 4 μm sections. Sections were rehydrated and blocked with serum. The sections were incubated with primary antibodies against ZO-1 (Abcam, England) and Occludin (Abcam, England) overnight at 4°C, followed by incubation with Alexa Fluor 488-conjugated secondary antibody (Beyotime Biotechnology, China) for 1 h at room temperature. Slides were stained with DAPI and immunofluorescence images were obtained using confocal microscopy (SP8, Leica). Quantification of images was determined by ImageJ software (National Institutes of Health, United States), to calculate the mean fluorescence intensity (MFI) per field.

### 2.7 RNA extraction and real-time PCR

Total RNA was extracted from intestine using the TRIzol reagent (Thermo Fisher Scientific, United States) and gauged by a Nanodrop spectrophotometer (Thermo Fisher Scientific, United States). RNA was reverse-transcribed (RT) using a Primescript RT reagent kit (Takara, Japan). mRNA levels were quantified by SYBR Green based on a Real-Time PCR System (ABI7500 Analyzer, Thermo Fisher Scientific, United States). Quantification was calculated using the 2-ΔΔCT method. The result is presented as fold change. β-actin was used as an internal control. Primers used in this study are as follows: ZO-1 (Forward: 5’-GCCGCTAAGAGCACAGCAA-3′; Reverse: 5’-GCCCTCCTTTTAACACATCAGA-3′); Occludin (Forward: 5’-TGAAAGTCCACCTCCTTACAGA-3′; Reverse: 5’-CCGGATA AAAAGAGTACGCTGG-3′); β-actin (Forward: 5’-GGCTGTAT TCCCCTCCATCG-3′; Reverse: 5′-CCAGTTGGTAACAATGCCA TGT-3′).

### 2.8 FITC-dextran assay

After myopia modeling success, food and water were withdrawn from all mice overnight. FITC-dextran (4 kDa, 600 mg/kg, 78,331, Sigma) was administered by a 20 G intragastric gavage needle at a concentration of 40 mg/mL in PBS. Four hours later, blood was collected and plasma was separated by centrifugation at 12,000×*g* for 10 min at 4°C. 200 μL supernatant of each sample was analysed on a plate reader at excitation/emission wavelengths of 485 nm/535 nm.

### 2.9 Statistics

Statistical analyses were performed using GraphPad Prism version 8, R (v3.3.2). Normally distributed data were used a two-tailed t test (unpaired) to compare the differences between the two groups, and the Wilcoxon rank-sum test was used if the variables were inconsistent with the normal distribution. *p* value of <0.05 was considered significant. Spearman’s correlation *p* values were corrected for multiple comparisons using the Benjamini-Hochberg false discovery rate.

## 3 Results

### 3.1 Construction a mouse model of high myopia

**Figure 1:**
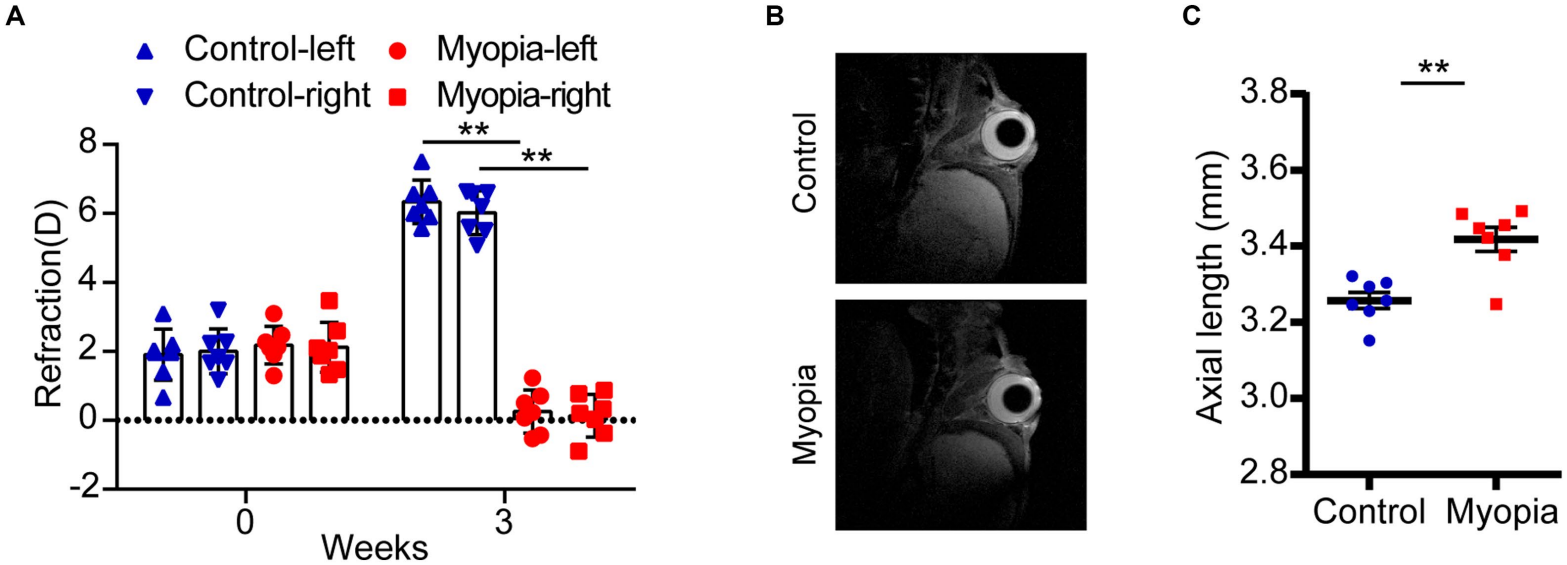
Myopic shift in a mouse model of myopia. **(A)** The refraction of eyes in control and myopic mice. **(B)** The representative image of MRI in control and myopic mice. **(C)** The axial length in control and myopic mice. Control group (*n* = 7), myopia group (*n* = 7). *p < 0.05, **p < 0.01.

Lens induced high myopia mice model was established in our study. The eyes of 3-week-old male mice were mounted with −30D lens for 3 weeks to induce high myopia. Mice with plain lens were used as control. The refractive diopter was evaluated by an infrared photorefractor. All the mice started with similar baseline diopters (Figure 1A). After 3 weeks, the diopters of myopia group were significantly lower than those in control group (Control-L vs. Myopia-L, 6.34 ± 0.63D vs. 0.25 ± 0.63D, *p* = 4.36E-10; Control-R vs. Myopia-R, 6.02 ± 0.63D vs. 0.13 ± 0.62D, *p* = 6.21E-10, Figure 1A). The axial length of eyes, measured by MRI, was significantly prolonged in myopic mice than controls (Control eyes vs. Myopia eyes, 3.26 ± 0.06 mm vs. 3.42 ± 0.08 mm, *p* = 0.0013, Figures 1B,C).

### 3.2 The α diversity and structure shift of the gut microbiome in myopic mice

**Figure 2:**
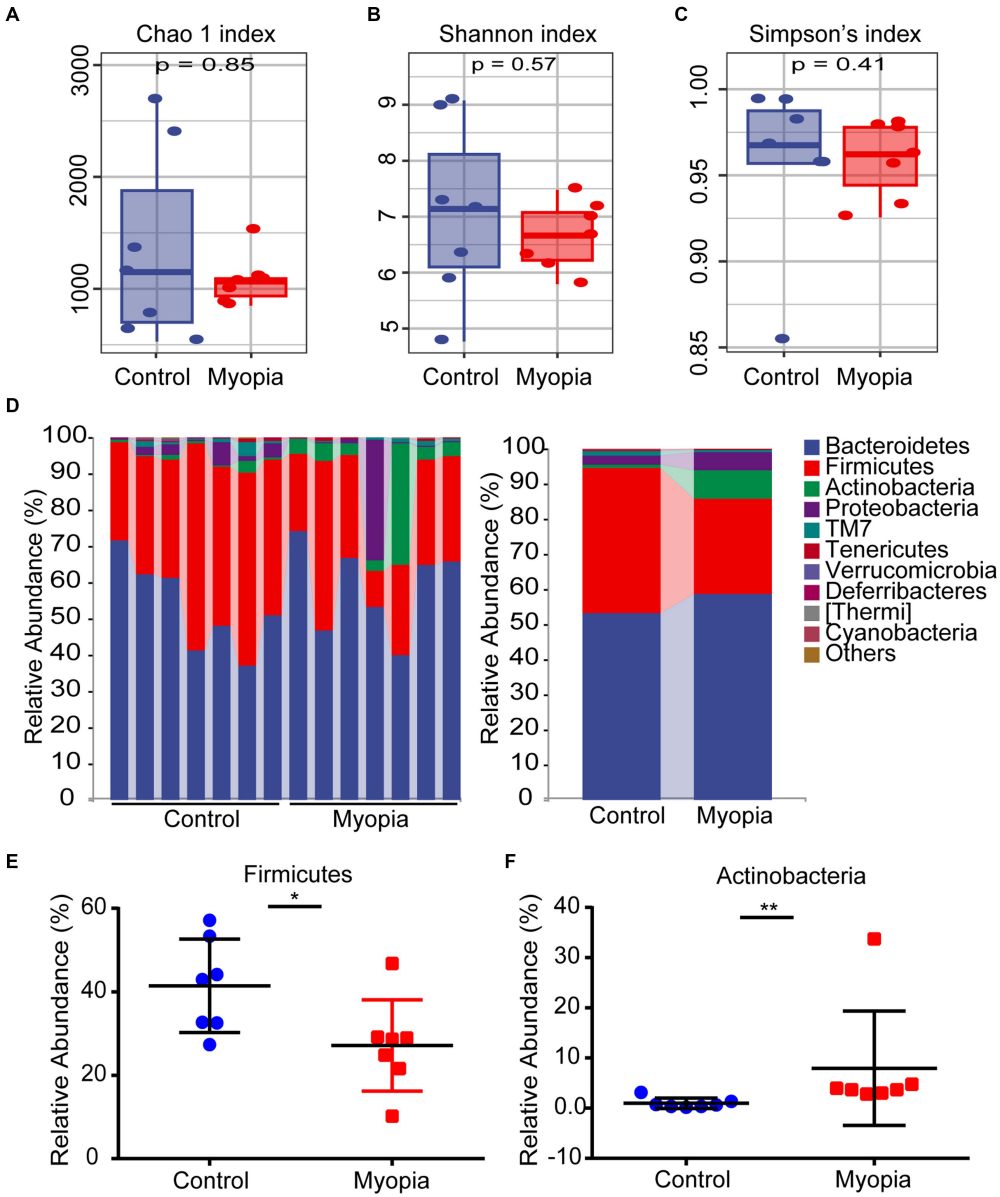
The α diversity and structure shift of the gut microbiome in myopic mice. Estimation of community by Chao 1 index **(A)**, Shannon index **(B)** and Simpson’s index **(C)** between the control and myopia groups. **(A–C)** The boxplots show median, 25th and 75th quartile. The Wilcoxon rank-sum test was used to analyze the statistical significance of alpha diversity. **(D)** Composition and relative abundance of bacterial phyla. Relative abundance of the Firmicutes **(E)** and Actinobacteria **(F)** phylum in myopia and control groups. Control group (*n* = 7), myopic group (*n* = 7). *p < 0.05, **p < 0.01.

We performed 16S rRNA gene sequencing to determine the alterations of gut microbiota composition in myopia. The Chao 1 index (Figure 2A), Shannon index (Figure 2B) and Simpson’s index (Figure 2C) showed no prominent difference between the control and myopia groups. These data declared that gut community α diversity remained unchanged in myopia.

As indicated by the plots of microbiota composition in Figure 2D, the microbial community structures were obviously altered between the control and myopia groups. Bacteroidetes, Firmicutes and Actinobacteria were the dominant phyla in the mouse’s gut microbiome (Figure 2D). Notably, after myopia induction, the relative abundance of Firmicutes was remarkably decreased from 41.43% to 27.15% (Figure 2E), while the relative abundance of Actinobacteria was significantly increased from 0.97% to 7.95% (Figure 2F).

### 3.3 Microbiome dysbiosis in myopia correlated with refractive diopter and AL

**Figure 3:**
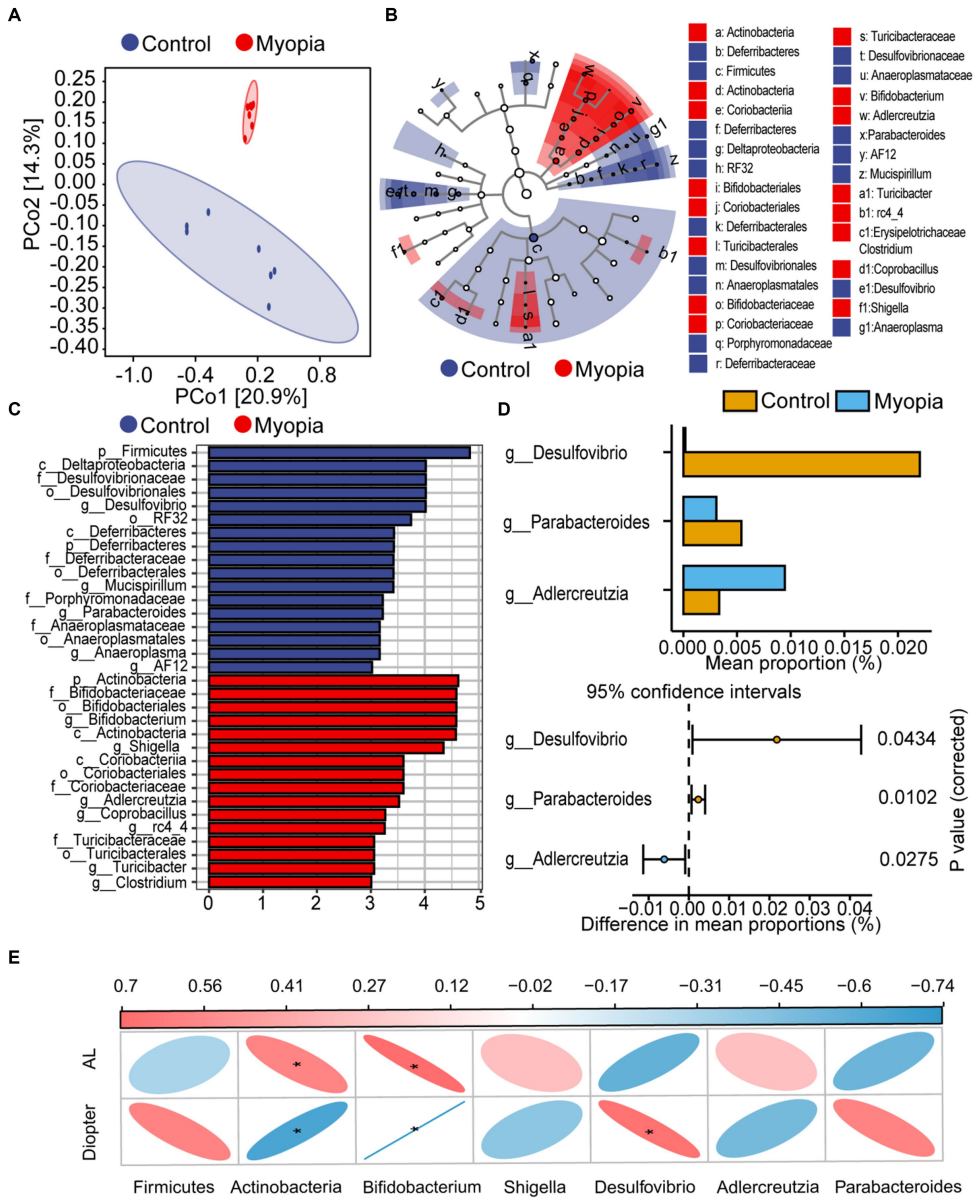
Microbiome dysbiosis in myopia correlated with refractive diopter and AL. **(A)** PCoA score plots based on unweighted UniFrac distance metrics (β-diversity) in the myopic and control mice. **(B)** Cladograms generated by LEfSe analysis indicate different taxonomic levels between control and myopic groups (LDA > 2.0). Blue circle indicates taxonomic enrichment in control mice, and red circle indicate taxonomic enrichment in myopic mice. **(C)** LDA scores (log10) of the bacterial taxa differentially abundant between myopia and control mice using LEfSe analysis. Blue bars indicate taxonomic enrichment in control mice, and red bars indicate taxonomic enrichment in myopic mice. **(D)** Statistical analysis of the LEfSe analysis variant gut microbiota in control and myopic mice by Student’s *t*-test. **(E)** Spearman’s correlation analysis of the correlation between differential bacteria and AL or diopter. Correlation coefficient is on the top side. Red indicates positive correlation and blue indicates negative correlation. Control group (*n* = 7), myopic group (*n* = 7). *FDR < 0.05, **FDR <0.01.

Beta diversity was visualized by PcoA diagram. PcoA analysis based on weighted UniFrac distance metrics confirmed the major bacterial composition was distinct between myopia and control mice (PERMANOVA, *R*^2^ = 0.14, *p* = 0.002, Figure 3A). To identify the biological characteristics of the dominant bacteria, linear discriminant analysis effect size (LEfSe) analysis was applied. The LDA distribution diagram (LDA > 2) showed obvious variations of the microbial DNA composition in myopic mice compared with the controls (Figure 3B). Interestingly, Bifidobacterium, Shigella and Adlercreutzia, regarded as pathobionts deteriorates gut barrier function (Bootz-Maoz et al., 2022; Leibovitzh et al., 2022), were particularly dominant genus in myopic mice according the LEfSe analysis (Figure 3C). To clarify the differences in microbiota abundance between the two groups, standard statistical analysis (Mann–Whitney test) was performed at the genus levels on the relative abundance of each taxon that was identified by LEfSe. The abundance of Desulfovibrio and Parabacteroides were prominently decreased while the abundance of Adlercreutzia was observably increased in myopic mice (Figure 3D). These initial findings revealed the gut microbiota structure shifted in the progression of myopia in lens-induced myopia mice model.

Spearman correlations were used to evaluate the associations between the abundance of bacterial taxonomic groups and severity of myopia. AL consistently showed a positive correlation with Actinobacteria and Bifidobacterium taxa (Figure 3E). Refractive diopter was positively correlated with Desulfovibrio taxa and negatively correlated with Actinobacteria and Bifidobacterium taxa. These findings suggested that the imbalanced composition of the microbiota participated in the myopia progression.

### 3.4 Plasma metabolomic profiling in myopic mice

**Figure 4:**
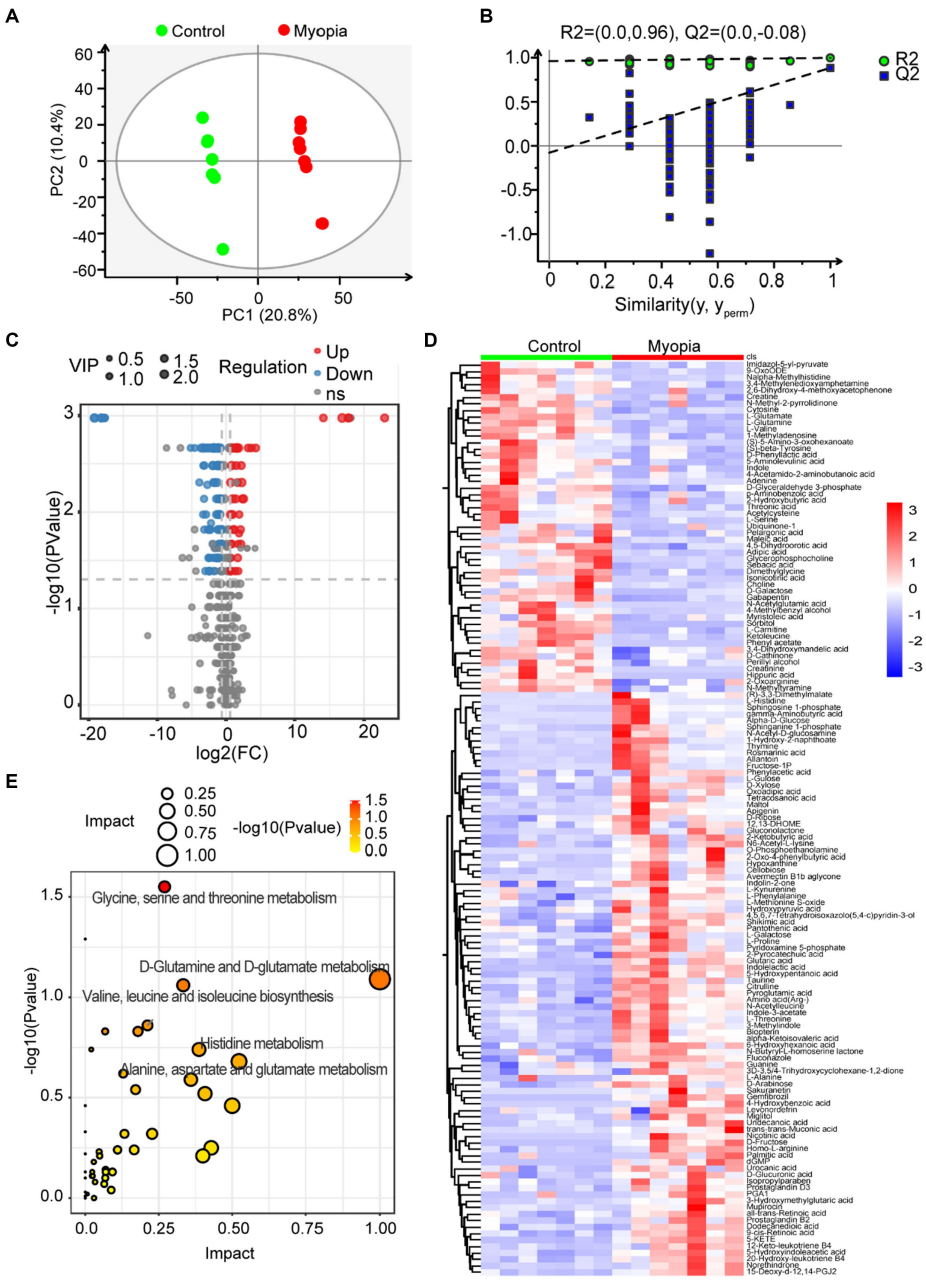
Serum metabolomic profiling in myopic mice. **(A)** The clustering analysis of partial least square discriminant analysis (PLS-DA) in control and myopic mice. **(B)** PLS-DA model test chart showed good discrimination between control and myopic mice. **(C)** The volcano plot of differential metabolites in control and myopic mice. **(D)** Hierarchical cluster analysis of differential metabolites between control and myopic mice. **(E)** The impact and *p* value of 141 significantly different metabolites involved in KEGG pathways across myopic and control mice. Control group (*n* = 7), myopic group (*n* = 7). *p < 0.05 and **p < 0.01.

To elucidate the relationship between the dysregulated microbiome in myopia and the host’s circulating metabolites, we employed non-targeted metabolomics to characterize plasma metabolomic profiles. We conducted PLS-DA to distinguish metabolic profiles among groups. The plasma metabolome showed a distinct clustering according to the PLS-DA plots by negative ionization mode (Figure 4A) and positive ionization mode (Supplementary Figure S1A). Goodness-of-fit values and predictive ability values by negative ionization mode (R2X = 0.312, R2Y = 0.998, Q2 = 0.884, Figure 4B) and positive ionization mode (R2X = 0.293, R2Y = 0.998, Q2 = 0.852, Supplementary Figure S1B) hinted that the PLS-DA model possessed a satisfactory fit with effective predictive power. Overall, 90 metabolites were upregulated and 51 metabolites were downregulated in myopic mice compared with the control mice (Figures 4C,D), indicating that myopia progression changed the components of the plasma metabolome.

To identify metabolic pathways that potentially play a role in myopia, we analyzed the pathway enrichment based on KEGG database. As shown in Figure 4E, the altered metabolites mainly involved in “Glutamine and D-glutamate metabolism,” “Alanine, aspartate and glutamate metabolism,” “Glycine, serine and threonine metabolism,” “Valine, leucine and isoleucine biosynthesis” and “Histidine metabolism” pathways, which all related to amino acid metabolism.

### 3.5 Associations of gut microbiota, circulating metabolites and myopia severity

**Figure 5:**
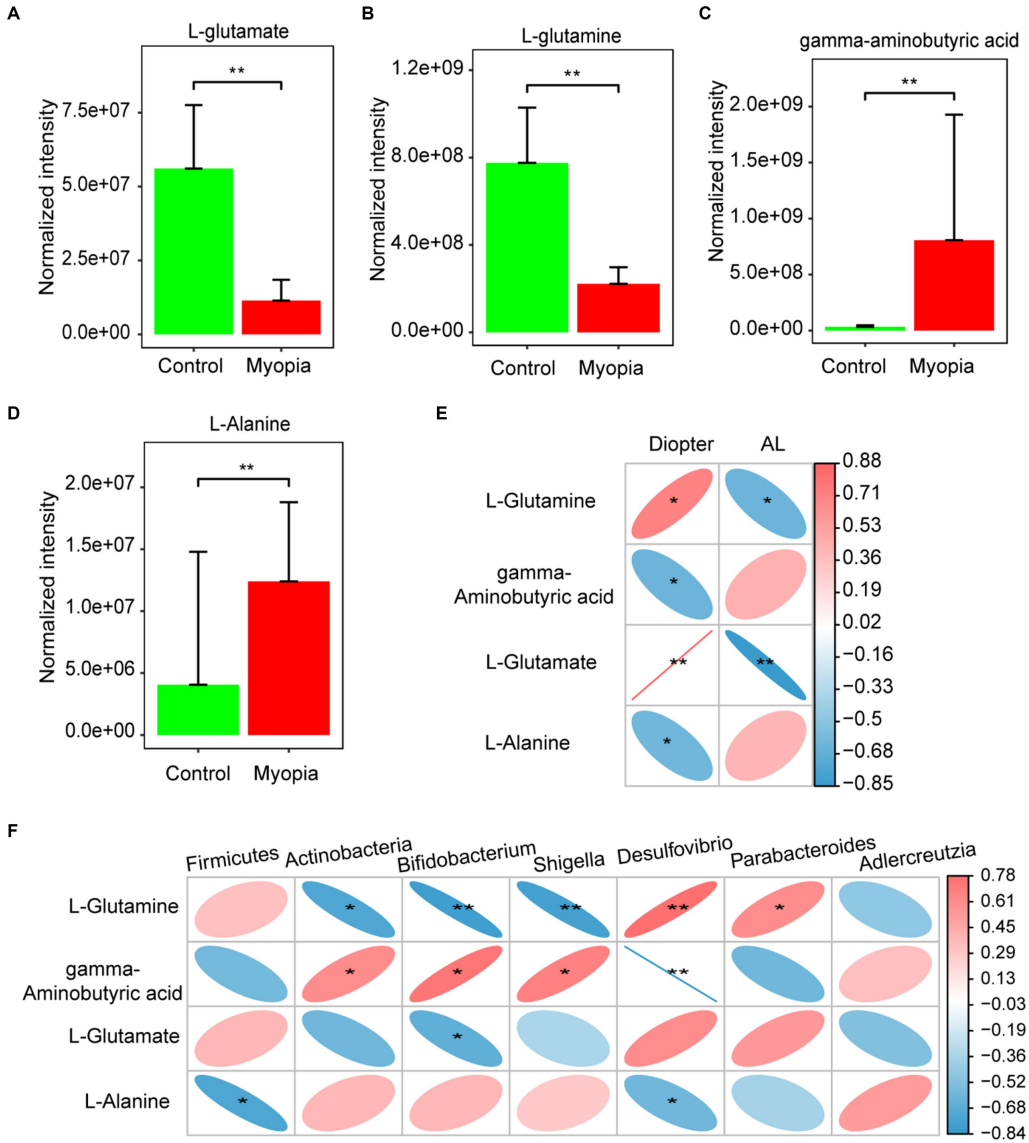
Associations of gut microbiota, circulating metabolites and myopia severity. The normalized intensities of L-glutamate **(A)**, L-glutamine **(B)** gamma-aminobutyric acid **(C)** and L-Alanine **(D)** were shown in myopia and control mice. **(E)** Correlation between differential metabolites involved in the glutamate metabolism and AL or diopter. **(F)** Correlation between differential bacteria and metabolites involved in the glutamate metabolism. **(E,F)** Correlation coefficient is on the right side. Red indicates positive correlation and blue indicates negative correlation. Control group (*n* = 7), myopia group (*n* = 7). *FDR <0.05, **FDR <0.01.

Interestingly, the most remarkable down-regulated pathway was related to the metabolism of glutamine and glutamate. We then analyzed the concentration variations of metabolites involved in glutamate metabolism. The normalized intensities of L-glutamate (Figure 5A) and L-glutamine (Figure 5B) were markedly decreased in myopic mice, while the normalized intensities of gamma-aminobutyric acid (Figure 5C) and L-Alanine (Figure 5D) were increased. Spearman’s correlation coefficient was calculated to determine the functional correlations between the changes in the metabolites and severity of myopia. The results showed that L-glutamate and L-glutamine were significantly correlated with refractive diopter positively, while significantly correlated with AL negatively. Gamma-aminobutyric acid and L-alanine levels were negatively correlated with refractive diopter (Figure 5E).

In addition, we identified the correlations between the changed metabolites and gut microbes in myopia. It is noteworthy that L-Glutamine was found to have a negative correlation with Actinobacteria, Bifidobacterium, and Shigella taxa, but a positive correlation with Parabacteroides taxa. Meanwhile, gamma-aminobutyric acid concentration was positively correlated with Actinobacteria, Bifidobacterium, and Shigella taxa, but negatively correlated with Desulfovibrio. L-Glutamate had a negative correlation with Bifidobacterium. Additionally, L-Alanine was negatively correlation with Firmicutes and Desulfovibrio (Figure 5F). Studies have highlighted the critical role of glutamate metabolism in the maintenance of mucosal integrity. Consistently, the dysbiosis in myopia have an interrupting gut barrier feature. Hence, the interrupting gut barrier feature of plasma metabolomics is accordant with the dysbiosis in myopia.

### 3.6 The gut barrier function was impaired in myopic mice

**Figure 6:**
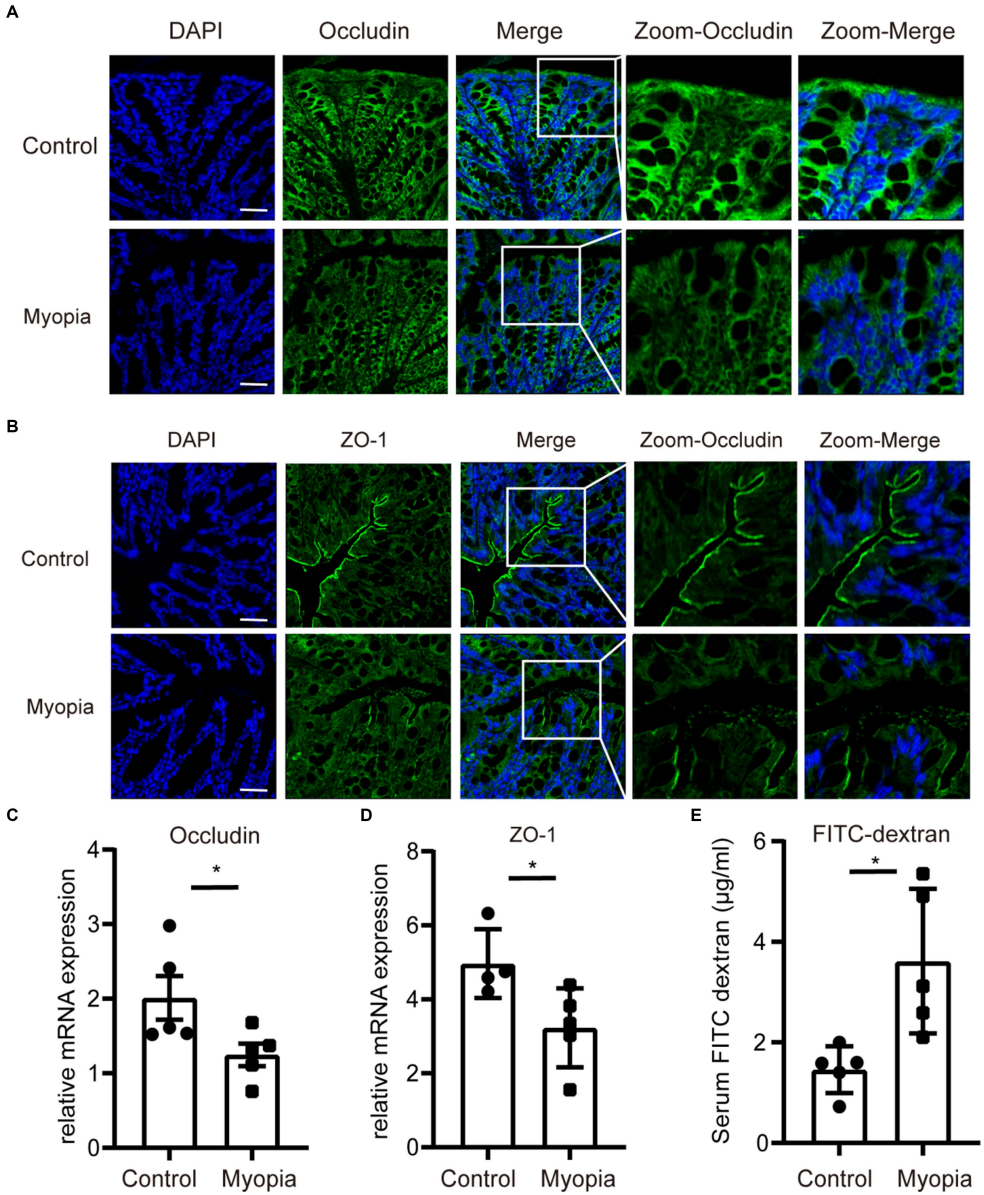
The gut barrier function was impaired in myopic mice. Immunofluorescence staining of Occludin **(A)** and ZO-1 **(B)** of the intestine in myopic and control mice. Scale bar = 200 μm. The relative mRNA level of Occludin **(C)** and ZO-1 **(D)** of the intestine in myopic and control mice. **(E)** FITC-dextran levels in the serum of myopic and control mice. Control group (*n* = 5), myopia group (*n* = 5). *p < 0.05, **p < 0.01.

Disorganisation of the microbial composition often leads to dysfunction of the gut mechanical barrier and immunological barrier. The disruption of intestinal barriers leads to an increase in inflammatory factors. Meanwhile, previous study found that myopia is associated with elevated levels of inflammatory marker. Therefore, we investigated the tight junction integrity and intestinal barrier function in myopic mice. Immunohistochemistry of intestinal tight junction proteins Occludin and ZO-1 showed decreased levels in myopic mice compared to controls (Figures 6A,B, Supplementary Figure S2A,B). Similarly, myopia progression remarkably decreased the mRNA level of Occludin and ZO-1 (Figures 6C,D) of the intestine. In addition, permeation of FITC-dextran of the gut in myopic mice was significantly incremented (Figure 6E). These findings illustrate a role for gut microbiota in directing mucosa barrier functions in myopia.

## 4 Discussion and conclusion

### 4.1 The shift of the gut microbiome in myopic mice was correlated with the severity of myopia

Host-microbiota interactions are crucial for host physiology and the disease phenotype (Peng et al., 2023). Dysbiosis may contribute to the development of diseases by increasing the presence of pathogens and their associated metabolites (Agus et al., 2021; Zhou et al., 2023). However, the role of microbiota in myopia remains unclarified. Herein, we integrated microbiota sequencing and metabolomics to investigate the microbiota and metabolites changes in myopia using a mouse model of lens-induced myopia.

Structure and functional changes in the commensal gut microbiota are thought to be involved in the pathogenesis of many diseases (Parker et al., 2022; Qiao et al., 2023). Several studies have elucidated that the bidirectional communication between the eye diseases and the gastrointestinal microbiota, indicating an accurate characterization of the gut microbiome is of critical importance for maintaining ocular health. In our study, we firstly explored the possible changes in the gut microbiome in myopia. We identified that Firmicute phylum was significantly reduced while Actinobacteria phylum was increased in myopia mice. Firmicutes, one of the major phyla in human gut microbiota, is essential for maintenance the integrity of the intestinal epithelial barrier, immune defenses against pathogenic microorganisms (Jandhyala et al., 2015; Lv et al., 2023). Firmicutes depletion is involved in diseases with impaired intestinal barrier including irritable bowel syndrome (Chen et al., 2023), colitis (Boger-May et al., 2022) and Crohn’s disease (Lv et al., 2023). On the other hand, Actinobacteria phylum contains numerous potential pathobionts that have the potential to cause intestinal inflammation (Daniel et al., 2021). In colitis, increased abundance of the phyla Actinobacteria invades the inner mucus layer, which mediates the aggravation of colitis (Boger-May et al., 2022). At genus level, Bifidobacterium and Shigella were particularly dominant genus in myopic mice. Previous studies have suggested that these elevated bacteria in myopia were pernicious bacteria, which were linked to negative health consequences. Bifidobacterium-derived exopolysaccharide (EPS) accumulated on the small intestinal villi disrupts the structure of the epithelial brush border (Sookoian et al., 2020) and induces differentiation of inflammatory immune response in Treg/Th17 axis (Yu et al., 2019). Oral gavage of *Bifidobacterium adolescentis* remarkably increases the inflammatory cytokines in small intestine (Bootz-Maoz et al., 2022). Moreover, Shigella is a genus of Gram-negative enteropathogens and its virulence factors could exacerbate gut barrier (Schnupf and Sansonetti, 2019). Together, these results suggested that the state of microbial dysbiosis in myopia is associated with inflammatory tendency and gut barrier disruption.

We further demonstrate that inflammation-related taxa, Actinobacteria and Bifidobacterium, are positively correlated with AL in the mouse model of myopia. Previous studies found an increased prevalence of myopia in inflammatory diseases such as diabetes (Meek et al., 2015), systemic lupus erythematosus (SLE) (Wang et al., 2019), uveitis and allergy (Lin et al., 2016). Indicators of systemic inflammatory properties, such as the neutrophil-to-lymphocyte ratio and platelet-to-lymphocyte ratio, are significantly elevated in patients with high myopia (Wang X. et al., 2022). Cyclosporine A treatment inhibits myopia progression by reducing the levels of inflammatory cytokines in the eye (Lin et al., 2016). Therefore, the progression of myopia may be influenced significantly by inflammation (Tien et al., 2021). Our results support that proinflammatory dysbiosis phenotype may be associated with myopia development. Therapeutic approaches targeting gut flora could be considered for the treatment of myopia.

### 4.2 Myopia induction affected the plasma metabolomic profiling mainly involved in glutamine and glutamate metabolism

The main way in which the gut microbiota interacts with the host is through metabolites, which are small molecules produced during microbial metabolism as intermediate or final products. Alterations have been described in the metabolite profiles in eye diseases. In our study, the composition of the microbial metabolome differed significantly between myopic and control mice. The differentially expressed metabolites were primarily enriched in “Glutamine and D-glutamate metabolism” pathways. Glutamine, the most abundant non-essential amino acid in the human body, which serves as a nutrition supplement and a neurotransmitter, could be metabolize into glutamate. Glutamine and glutamate metabolic processes are essential in eye development (Pletcher et al., 2019). Glutamine concentration varies in retinopathy (Xia and Zhang, 2022) and dry eye disease (Quartieri et al., 2021). As for glutamate, it has been identified as the major neurotransmitters in the radial and lateral synaptic pathways of the vertebrate retina (Barnstable, 1993). The retina has an important role in the signal transmission related to eye development and myopia (Guoping et al., 2017). In the current study, the concentration of glutamine and glutamate were negatively associated with the severity of myopia, indicating that intestinal microbiota related amino acid metabolites might be linked to myopia. Further investigation is required to understand the effects of decreased glutamine and glutamate levels in myopia.

### 4.3 The intestinal barrier in myopic mice was disrupted, which was consistent with the interrupting gut barrier feature of dysbiosis and plasma metabolomics shift in myopic mice

Recent studies highlighted a critical role for glutamine metabolism in the maintenance of mucosal integrity. In gut physiology, glutamine and glutamate promote enterocyte proliferation, maintain intestinal barrier function and limits intestinal inflammation (Tome, 2018; Hu et al., 2019). Dysfunction of the mucosal barrier is associated with increased circulating inflammatory cytokines and development of multiple inflammation-related diseases (Shin and Kim, 2018). As mentioned above, inflammation may play a vital role in the progression of myopia. The levels of IL-6, MMP-2, and TGF-β in the aqueous fluid of highly myopic eyes were higher than those in non-highly myopic eyes (Zhu et al., 2016; Yuan et al., 2019). Additionally, the dysbiosis and plasma metabolomics shift in myopia have an interrupting gut barrier feature. We further investigated the gut barrier function and found gut tight junction was impaired in myopic mice. Numerous studies have shown that various diseases and disorders are linked to a dysfunctional intestinal barrier. For example, Alzheimer’s disease mice exhibited a compromised epithelial barrier and ongoing inflammation in both the intestines and throughout the body (Pellegrini et al., 2023). Transplantation of the faecal from healthy mice can restore intestinal barrier and attenuate cognitive impairment (Kim et al., 2020). Shifts in gut microbiota and increased gut permeability in myocardial infarction patients can lead to elevated systemic inflammatory factors, thereby exacerbating the symptoms of myocardial infarction (Zhao et al., 2023). Therefore, dysbiosis of gut microbiota and disruption of intestinal barrier in myopic mice may promote the progression of myopia by increasing systemic inflammation level.

## 5 Limitations and conclusion

Our research has certain limitations. We conducted our study using an animal model, and the highly myopic state induced by lens in a short term may not fully represent the actual development of myopia in humans. Therefore, further studies are needed to verify the gut microbiota changes in myopic patients.

In conclusion, this study revealed that myopic mice had obviously altered gut microbiome and metabolites profiles compared to non-myopic mice. The dysbiosis and plasma metabolomics shift in myopia have an interrupting gut barrier feature. Our study provides novel insights into the mechanisms underlying the comorbidity of myopia and inflammation response. Targeting these microbes or their metabolites could be a promising new strategy for alleviating myopia progression.

## Data availability statement

The data presented in the study are deposited in the Sequence Read Archive (SRA) with the accession number PRJNA1001840 and MetaboLights repository with the accession number MTBLS9006.

## Ethics statement

Animal experiments in this study were approved by the Ethics Committee for Animal Studies of Eye & ENT Hospital of Fudan University. The study was conducted in accordance with the institutional requirements.

## Author contributions

HL and YL: conceptualization. HL: methodology, software, formal analysis, investigation, data curation, writing-original draft preparation, and visualization. HL, SL, and KZ: validation. XZ, JD and YL: resources, supervision, project administration, and funding acquisition. HL and SL: writing-review and editing. All authors contributed to the article and approved the submitted version.

## Footnotes

^1^ www.metaboanalyst.ca
