# Supplementary material for: Gut microbiome and plasma metabolome alterations in myopic mice
Source: Front Microbiol. 2023 Dec 21;14:1251243. doi: 10.3389/fmicb.2023.1251243 (PMC10764480; doi:10.3389/fmicb.2023.1251243)
Supplement: Supplementary file 1 [file Data_Sheet_1.docx]

Supplementary Material

##
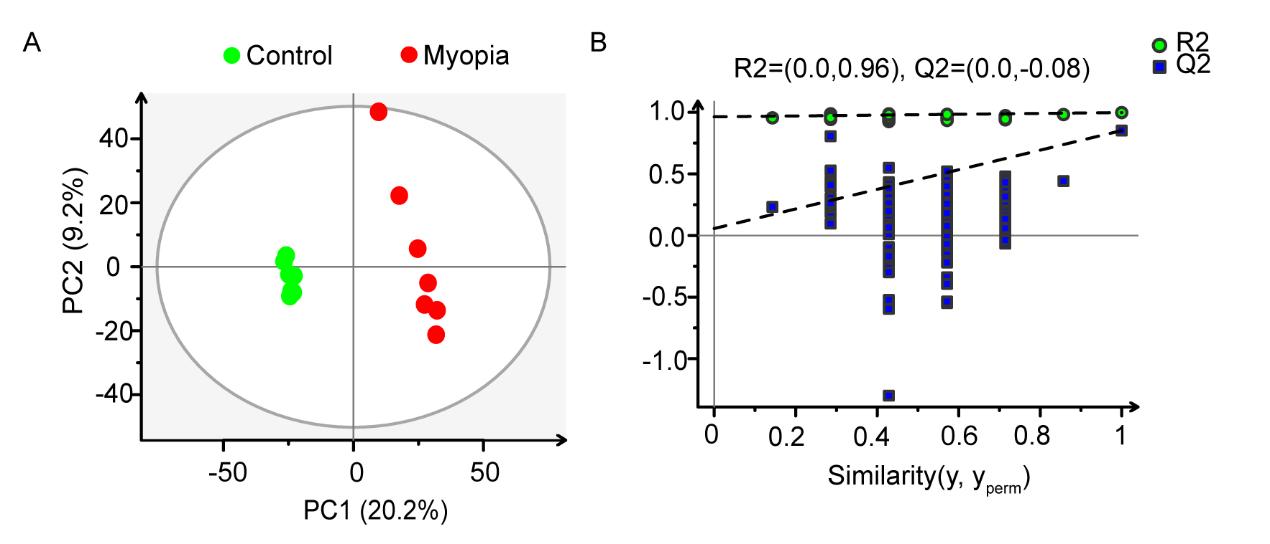
Supplementary Figures

**Supplementary Figure 1. PLS-DA and PLS-DA model test by positive ionization mode**


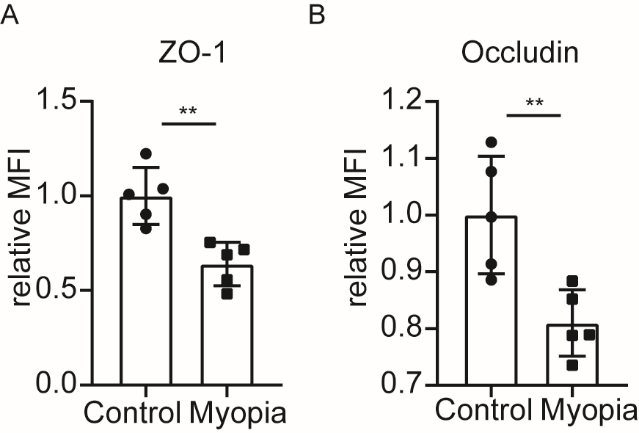
(A) The clustering analysis of PLS-DA in control and myopic mice by positive ionization mode. (B) PLS-DA model test chart showed good discrimination between control and myopic mice by positive ionization mode.

**Supplementary Figure 2. Immunofluorescence analysis of ZO-1 and Occludin protein expression in** **intestine.**

(A) The relative MFI of ZO-1 protein expression in intestine. (B) The relative MFI of Occludin protein expression in intestine.
